# First-Principles Discovery of Novel LiInP_2_S_6_ Polymorphs with Promising Optoelectronic Responses

**DOI:** 10.1021/acs.jpcc.5c08009

**Published:** 2026-03-17

**Authors:** Pegah Mohammadi, Arjyama Bordoloi, Sobhit Singh

**Affiliations:** † Department of Mechanical Engineering, 6927 University of Rochester, Rochester, New York 14627, United States; ‡ Material Science Program, 6927 University of Rochester, Rochester, New York 14627, United States

## Abstract

Recent advances in two-dimensional (2D) van der Waals (vdW) metal thiophosphates have attracted considerable attention due to their promising ionic conductivity, optical characteristics, and tunable physical properties. Within this material family, LiInP_2_S_6_ has emerged as an intriguing candidate, not only because of its sensitivity to air and moisture but also due to its suitable band gap within the UV–vis range, enabling potential optoelectronic and photocatalytic applications. In this study, through comprehensive first-principles investigations, we unveil two previously unreported polymorphs of LiInP_2_S_6_ in the monoclinic *C*2/*c* and trigonal *P*3̅1*c* (in-gap) space groups, in addition to examining the experimentally synthesized *P*3̅1*c* (in-layer) phase. Our studies identify the *C*2/*c* structure as the ground state, lying 9 meV per unit cell lower in energy than the experimentally realized trigonal *P*3̅1*c* (in-layer) phase. Further, we systematically examine the elastic, mechanical, thermodynamical, dynamical, electronic, and optical properties of all three polymorphs, confirming their mechanical, thermal, and dynamical stability. Notably, the *P*3̅1*c* (in-gap) phase exhibits enhanced stiffness, while the calculated indirect band gaps and strong photon absorption in the UV–vis range (∼3 eV) highlight the potential of the studied LiInP_2_S_6_ phases for iontronic devices and optoelectronic applications.

## Graphical abstract

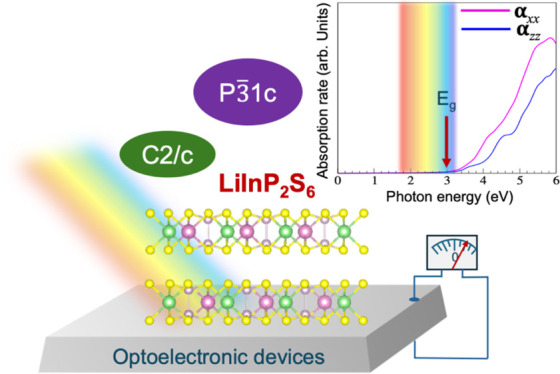

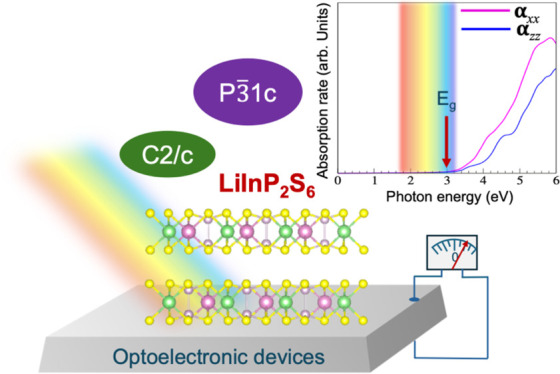

## Introduction

Recently, two-dimensional van der Waals (vdW) materials have attracted significant attention owing to their strong intralayer covalent bonding and weak interlayer interactions, which together yield a high surface-to-volume ratio and tunable surface properties favorable for ion transport.^1,2^ Among these, vdW-layered metal thiophosphates, M_*x*_P_2_X_6_ (M = metal, X = S or Se), are particularly notable for their high ionic conductivity.^3–7^ In this context, CuInP_2_S_6_ has been extensively investigated due to its remarkable ionic conductivity^3–7^ and room-temperature ferroelectricity observed in thin-film form.^8–17^ Recent works on AgBiP_2_Se_6_ reports a vdW-layered semiconductor with strongly anisotropic optical properties.^18,19^ Furthermore, LiInP_2_Se_6_, another closely related member of this materials family possesses the requisite properties for direct thermal neutron detection, highlighting its potential for radiation-sensing applications.^20,21^

In addition, the ongoing global industrialization has motivated the investigation of moisture-sensitive materials and has increased the demand for thiophosphates, which exhibit semiconductor-like resistive, capacitive, and optical responses.^22–25^ More recently, lithium thiophosphates (Li_*x*_M_*y*_P_2_S_6_), containing highly hydrophilic alkali metals, have demonstrated pronounced air and moisture sensitivity, further broadening their potential applications in areas such as waste treatment.^26^

Recently, the first experimental investigation of LiInP_2_S_6_ was reported by Liang et al.^27^ in 2024, who demonstrated a one-step intercalation–exfoliation method for synthesizing LiInP_2_S_6_ nanosheets. They characterized the material in the trigonal *P*3̅1*c* (no. 163) phase and revealed the spontaneous incorporation of water molecules into the LiInP_2_S_6_ crystal lattice, identifying it as a promising candidate for inorganic iontronic devices. In a subsequent study,^28^ they reported that exposure of LiInP_2_S_6_ to ambient air enables spontaneous intercalation of water molecules into the vdW gaps, forming a hydrated phase. This hydration process significantly enhances the ionic conductivity at room temperature, making the material a candidate for future superionic conduction applications.

Later, Qian et al.^29^ synthesized LiInP_2_S_6_ through both direct stoichiometric combination and dissolution in a reactive P_2_S_5_ flux, and investigated the optoelectronic properties and electronic structure of the resulting samples. More recently, Bai et al.^30^ studied the optoelectronic properties of monolayer LiInP_2_S_6_/XTe_2_ (X = Mo, W) vdW heterojunctions using first-principles calculations, revealing their potential applicability in next-generation optoelectronic devices. Collectively, these studies underscore the promise of LiInP_2_S_6_ for diverse functional applications. However, to the best of our knowledge, several structural polymorphs of LiInP_2_S_6_, including its ground-state structure, have not yet been systematically investigated, and their elastic, mechanical, electronic, and optical properties remain largely unexplored.

To address this gap, in this work, we employ first-principles density-functional theory (DFT) calculations to design and investigate several new two-dimensional LiInP_2_S_6_ vdW polymorphs; the monoclinic *C*2/*c*, and the trigonal *P*3̅1*c* (in-layer) and *P*3̅1*c* (in-gap) phases, as illustrated in Figure 1. Among these, the *C*2/*c* phase is identified as the ground-state structure, with a relative energy of 9 meV/u.c. less than the previously reported *P*3̅1*c* (in-layer) phase. We further explore the elastic, mechanical, thermal, dynamical, electronic, and optoelectronic properties of these three candidates. Our results reveal that all three phases are thermodynamically, dynamically, and mechanically stable, indicating their experimental feasibility. Moreover, their band gaps, lying near the UV–vis range (∼3 eV), motivate a detailed examination of their optical responses and potential applications in optoelectronic devices.

**Figure 1:**
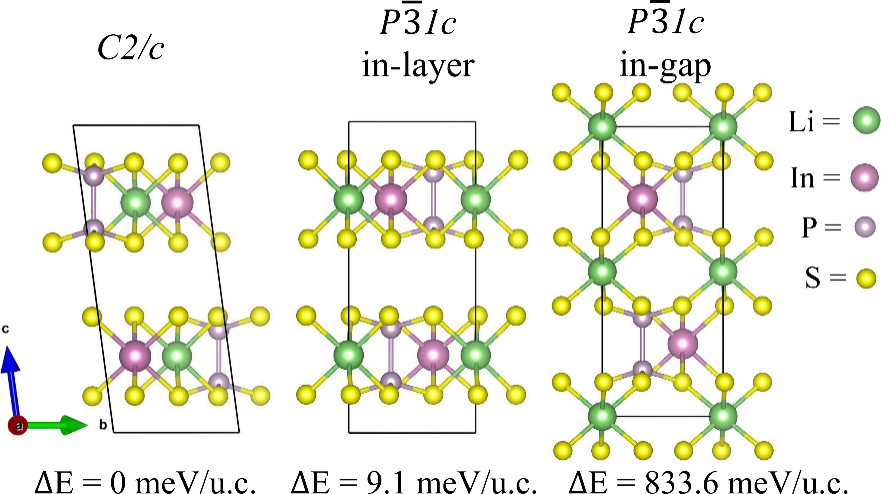
VdW-layered LiInP_2_S_6_ structures illustrating three different polymorphs: one monoclinic *C*2/*c* phase and two trigonal P3̅1c phases, distinguished by the Li atom residing either within the intralayer (i.e., in-layer) region or in the interlayer vdW gap (i.e., in-gap). Their corresponding energies are shown relative to the ground-state *C*2/*c* phase, which has a reference energy of −100.046 eV/u.c.

## Methods

The reported DFT calculations were carried out using the Vienna *Ab-initio* Simulation Package (VASP) with the projector augmented wave (PAW) method,^31–34^ employing the generalized gradient approximation (GGA) to the exchange-correlation functional, as parametrized by Perdew–Burke–Ernzerhof for solids (PBEsol).^35^ Weak vdW interactions were considered using the DFT-D3 method.^36,37^ The force convergence criterion for atomic relaxation was set to 10^–3^ eV/Å, and the total self-consistent energy was converged to 10^–7^ eV. Brillouin zone sampling was performed using a Γ-centered 8 × 8 × 4 k-point mesh. In the PAW pseudopotentials, contributions from one valence electron for Li (2s^1^), three for In (2s^2^2p^1^), five for P (3s^2^3p^3^), and six for S (3s^2^3p^4^) were included.

The dynamical stability of the studied systems was assessed via phonon calculations in 2 × 2 × 1 supercells along with a 4 × 4 × 4 k-mesh using the finite displacement method as implemented in VASP. Postprocessing of phonon data was performed using the Phonopy.^38^ In addition, the LO-TO splitting was accounted for by applying a nonanalytical term correction.^39–41^ The elastic constants *C*_*ij*_ were converged by refining the k-point mesh. A detailed investigation of the elastic properties, including the evaluation of elastic wave velocities and Debye temperature, was conducted using the MechElastic Python package.^42,43^ The thermodynamic stability of the structures was examined using *ab initio* molecular dynamics (AIMD) simulations carried out within the canonical (NVT) ensemble using a Nosé–Hoover thermostat at 300 K, with a total simulation time of 10 ps.^44^ The electronic structure was analyzed using the Pyprocar software.^45,46^ The electronic and optical properties were further examined using the HSE06 hybrid functional.^47^ The frequency-dependent optical properties were calculated using the LOPTICS = .TRUE. implementation in the VASP package.^48^ Within this independent-particle formalism, the complex, frequency-dependent dielectric function is obtained from interband transitions between occupied and unoccupied Kohn–Sham states. As the generalized gradient approximation typically underestimates the band gap, a scissor correction was applied to rigidly shift the unoccupied electronic states obtained from PBEsol to match the HSE06-computed band gap, thereby achieving improved agreement with experimental data.^49–51^ Postprocessing of the dielectric function and absorption coefficient was performed with the Vaspkit package.^52^

## Results and Discussion

### Structural Polymorphs

In this study, we investigate three candidate crystal structures of layered vdW LiInP_2_S_6_ material, as illustrated in Figure 1. Among them, we propose two previously unreported polymorphs: a monoclinic *C*2/*c* (no. 15) phase and a trigonal *P*3̅1*c* in-gap (no. 163) phase, where Li atoms reside within the vdW interlayer gap. In addition, we examine the experimentally synthesized trigonal *P*3̅1*c* in-layer (no. 163) phase, in which Li atoms reside within each monolayer.^27,28^ Besides, we considered several other polar candidate phases – such as *Cc* (no. 9) and *P*31*c* (no. 159), previously reported for the sister compound CuInP_2_S_6_ (see ref 16.). However, upon structural relaxation, all of these polar phases spontaneously relaxed into their corresponding nonpolar centrosymmetric ground-state structures shown in Figure 1.

After fully relaxing each structure, the obtained lattice parameters and relevant cell angles are listed in Table 1. (The corresponding fractional atomic coordinates for each prototype structure are provided in Table S1.) Their relative energetic stabilities are then evaluated based on the total energy comparisons. Our results reveal that the *C*2/*c* phase exhibits the lowest total energy, identifying it as the most energetically favorable configuration and the ground-state structure of this material. Meanwhile, the experimentally synthesized *P*3̅1*c* in-layer phase is a metastable structure, lying 9 meV/u.c. above the ground state phase. The primary structural differences between these two phases originate from their crystal symmetry, lattice geometry, and the relative positions of the Li ions. In the monoclinic *C*2/*c* phase, the lattice parameters satisfy *a* ≠ *b* ≠ *c*, with α = β = 90° and γ ≠ 90°, reflecting its lower symmetry. By comparison, the trigonal *P*3̅1*c* phase exhibits higher symmetry, characterized by *a* = *b* ≠ *c*, α = β = 90°, and γ = 120°. Consequently, the monoclinic *C*2/*c* phase represents a lower-symmetry yet energetically more stable configuration relative to the higher-symmetry trigonal *P*3̅1*c* phase.

**Table 1:** DFT (PBEsol+D3) Optimized Lattice Parameters and Relative Energies (Δ*E*) of the Three Studied Polymorphs^a^

Phase	*a* (Å)	*b* (Å)	*c* (Å)	α (°)	β (°)	γ (°)	Δ*E* (meV/u.c.)
*C*2/*c*	6.02	6.02	12.91	94.3	94.3	120.0	0.00
*P*3̅1*c* (in-layer)	6.02	6.02	12.60	90.0	90.0	120.0	9.1
Expt.^27^	(6.08)	(6.08)	(12.94)	90.0	90.0	120.0	
*P*3̅1*c* (in-gap)	6.05	6.05	12.72	90.0	90.0	120.0	833.6

a Energies are given relative to the monoclinic *C*2/*c* phase, taken as the reference with −100.04 eV/u.c. total energy. The experimental data for the *P*3̅1*c* in-layer phase from ref 27 are shown in parentheses.

In contrast, the *P*3̅1*c* in-gap phase has the highest total energy, lying 833 meV/u.c. above the *C*2/*c* ground-state structure. Despite its significantly higher energy, it exhibits full thermodynamical, dynamical, elastic, and mechanical stability, as discussed below. Furthermore, the thermodynamical stability of the two previously unreported *C*2/*c* and *P*3̅1*c* (in-gap) phases was confirmed by *ab initio* molecular dynamics simulations (AIMD) performed at 300 K for 10 ps (see Figure S1). These results verify that all three studied polymorphs are thermodynamically stable. The thermodynamical stability of the *P*3̅1*c* (in-layer) phase is also supported by its experimental synthesis at room temperature.^27,28^

### Elastic and Mechanical Properties

The macroscopic mechanical properties of materials, such as Young’s modulus (*E*), Poisson’s ratio (ν), bulk modulus (*K*), and shear modulus (*G*), are critical indicators of their elastic behavior and mechanical stability. A comprehensive understanding of these properties is essential for evaluating the potential of materials for various technological applications.

To analyze these mechanical properties, it is important to introduce the elastic stiffness constants *C*_*ijkl*_ and establish their relationship with the macroscopically measurable quantities. This relationship can be derived using the generalized Hooke’s law,^54^ as expressed by the following relation:

$$\sigma_{ij}=C_{ijkl}\varepsilon_{kl}$$ 1

Here, σ_*ij*_ and *ε*_*kl*_ represent the homogeneous second-rank stress and strain tensors, respectively, and *C*_*ijkl*_ denotes the fourth-rank elastic stiffness tensor.

The relevant elastic constants obtained from the MechElastic package^43^ are listed in Table 2. To assess the mechanical stability of the studied phases, we apply the Born–Huang mechanical stability criteria.^54–56^ According to these criteria, a crystal is considered mechanically stable if its Gibbs free energy, in the absence of any external load, corresponds to a minimum with respect to all possible infinitesimal strains. This implies that the elastic stiffness matrix *C*_*ij*_ must be positive definite and symmetric. If a crystal, satisfies the set of Born–Huang stability conditions corresponding to its space group as outlined in ref. 43, it can be regarded as mechanically stable. In our study, all three considered phases satisfy the Born–Huang criteria and are thus considered mechanically stable. All six eigenvalues of the *C*_*ij*_ matrix are positive, indicating the elastic stability of proposed three phases.

**Table 2:** Elastic Stiffness Constants *C*_*ij*_ (in GPa) for the Studied LiInP_2_S_6_ Phases

Phase	*C*_11_	*C*_22_	*C*_33_	*C*_44_	*C*_55_	*C*_66_	*C*_12_	*C*_13_	*C*_14_	*C*_15_	*C*_23_	*C*_25_	*C*_35_	*C*_46_
*C*2/*c* (#15)	95.4	95.9	42.5	9.7	9.6	35.4	24.5	7.0	0.0	1.1	7.1	–1.1	–0.18	–1.1
*P*3̅1*c* (in-layer, #163)	100.0	100.0	37.2	9.3	9.3	37.2	25.5	6.4	0.0	–3.1	6.4	3.1	0.0	3.1
*P*3̅1*c* (in-gap, #163)	92.2	92.2	55.2	16.5	16.6	29.6	32.6	17.0	0.02	–1.1	17.0	1.0	0.0	1.0

Once the elastic constants *C*_*ij*_ are determined, the four fundamental elastic moduli (*K*, *E*, *G*, ν) can be derived based on their standard relationships with the elastic constants. The Voigt-Reuss-Hill (VRH) averaging scheme^58^ is first employed to calculate the bulk and shear moduli (*K* and *G*). Subsequently, ν and *E* are computed using their conventional definitions in terms of *K* and *G*, as implemented in the MechElastic package.^43^

The computed mechanical and elastic moduli for the studied LiInP_2_S_6_ candidates are summarized in Table 3. Based on the calculated mechanical properties, the *P*3̅1*c* in-gap phase exhibits the highest stiffness and is overall harder compared to the *P*3̅1*c* in-layer and *C*2/*c* phases. In the *P*3̅1*c* in-gap phase, the Li atoms occupy interlayer sites, enhances covalent bonding across the vdW gap, leading to increased mechanical strength. In contrast, when Li atoms are located within intralayer positions (*C*2/*c* and *P*3̅1*c* in-layer) the interlayer vdW interaction resulting in softer and more compressible behavior. Since all three studied phases exhibit a ν < 0.26, this indicates a predominantly ionic bonding character in the structure and suggests a brittle mechanical behavior.^59^

**Table 3:** Elastic Constants and Derived Mechanical and Thermodynamic Properties for LiInP_2_S_6_ Phases

Phase	*K* (GPa)	*G* (GPa)	*E* (GPa)	ν	*v*_l_ (m/s)	*v*_t_ (m/s)	*v*_m_ (m/s)	Θ_D_ (K)
*C*2/*c* (#15)	31.4	20.2	49.9	0.23	4331.8	2548.5	2824.6	309.4
*P*3̅1*c* (in-layer, #163)	30.6	20.0	49.2	0.23	4270.9	2522.8	2795.1	307.1
*P*3̅1*c* (in-gap, #163)	39.6	23.0	57.8	0.25	4766.5	2727.5	3030.4	331.4

To provide a broader perspective, we compare the elastic moduli of the studied LiInP_2_S_6_ polymorphs with those of a sister compound, LiInP_2_Se_6_, which has been experimentally synthesized in the *P*3̅1*c* (in-layer) phase and is a known member of the two-dimensional metal phosphorus trichalcogenide (MPTC) family with promising technological applications^57^ [see Figure 2]. Since the in-plane covalent framework is similar in LiInP_2_S_6_ and LiInP_2_Se_6_, their in-plane Young’s moduli are expected to be comparable. This expectation is consistent with the experimentally reported value of 51.2 ± 4.5 GPa for LiInP_2_Se_6_, as well as with our calculated value of 49.2 GPa for LiInP_2_S_6_.

**Figure 2:**
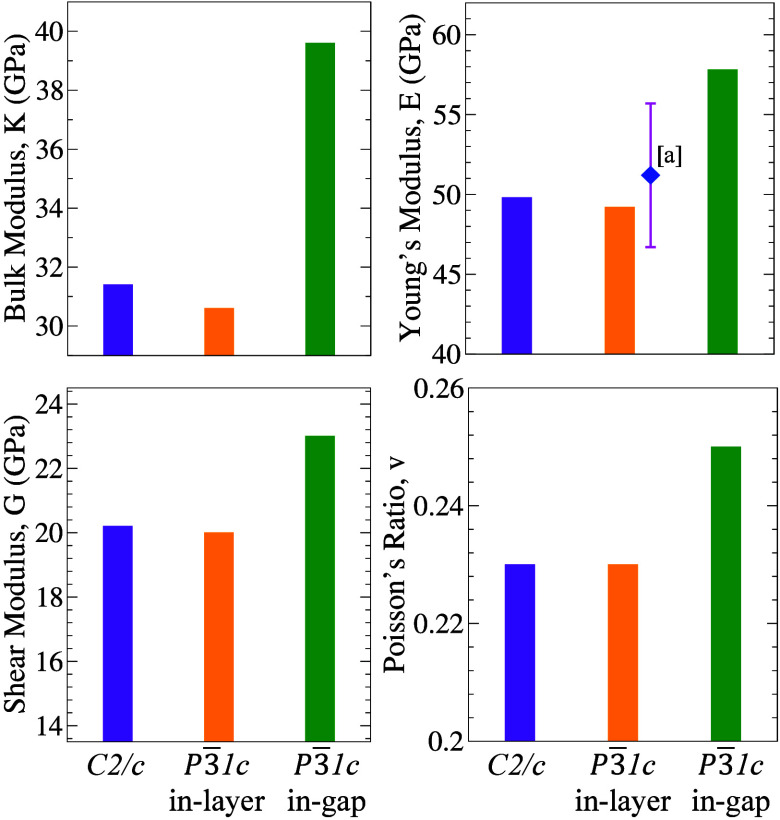
Elastic constants of LiInP_2_S_6_ in the *C*2/*c* and *P*3̅1*c* (in-layer and in-gap) phases compared with other materials. [a] The ◆ symbol denotes the experimental study^57^ on LiInP_2_Se_6_, reporting the Young modulus (*E*).

To assess the ductile or brittle nature of a material, the ratio of the bulk modulus to the shear modulus is evaluated. A *K*/*G* value greater than 1.7 typically indicates ductile behavior.^43^ In this study, all three investigated phases of LiInP_2_S_6_ exhibit values below this threshold, suggesting a brittle mechanical character. This is further verified by the corresponding Poisson’s ratio values, which are consistent with brittle behavior.

Next, for evaluating the experimental feasibility of LiInP_2_S_6_ polymorphs, we estimate their elastic wave velocities. The longitudinal (*v*_l_), transverse (*v*_t_), and average (*v*_m_) elastic wave velocities are obtained using the MechElastic package,^43^ based on the following relations:

$$v_{l}=\sqrt{\frac{3K+4G}{3\rho }}$$ 2

$$v_{t}=\sqrt{\frac{G}{\rho }}$$ 3

and

$$v_{m}={\left[\frac{1}{3}\left(\frac{2}{{v_{t}}^{3}}+\frac{1}{{v_{l}}^{3}}\right)\right]}^{-1/3}$$ 4

Here, ρ denotes the density of the material. By investigating calculated elastic wave velocities, the *P*3̅1*c* in-gap phase exhibits the highest longitudinal, transverse, and average sound velocities, indicating its higher stiffness than that of two other candidates.

The Debye temperature (Θ_*D*_) is an important thermodynamic parameter that provides deep insight into the lattice vibrational properties of solids. It is related to thermal behaviors, such as specific heat, thermal conductivity, and lattice stability, and thus serves as a valuable parameter of the strength of interatomic bonding within a material, and can be obtained from the average elastic wave velocity *v*_m_ and the density ρ. It is worth noting that, at low temperatures, Θ_*D*_ reflects from the acoustic phonons, which are the dominant vibrational excitations contributing to the specific heat. Therefore, the low-temperature Debye temperature estimated from the elastic constants is approximately equal to the value obtained from specific heat measurements.^60^ Θ_*D*_ is calculated using the following formula:

$$\theta_{\mathrm{Debye}}=\frac{h}{k_{B}}{\left[\frac{3q}{4\pi }\cdot \frac{N\rho }{M}\right]}^{1/3}v_{m}$$ 5

where, *h* is Planck’s constant, *k*_B_ is Boltzmann’s constant, *q* is the total number of atoms in the unit cell, *N* is Avogadro’s number, and *M* is the molecular weight. Consistently, the Debye temperature of the *P*3̅1*c* in-gap phase is the highest among the three phases, further confirming its strong interatomic interactions and stiffer vibrational lattice dynamics compared to the *C*2/*c* and *P*3̅1*c* in-layer phases. Since all the studied LiInP_2_S_6_ polymorphs are found to be elastically and mechanically stable, we now turn our attention to examining their and dynamical stability.

### Dynamical Stability

To evaluate the dynamical stability of all LiInP_2_S_6_ polymorphs, we calculate the full phonon spectra plotted along the high-symmetry directions of the Brillouin zone, as shown in Figure 3. The absence of imaginary phonon frequencies throughout the entire Brillouin zone for all three phases confirms their dynamical stability. This suggests that these structures are experimentally viable.

**Figure 3:**
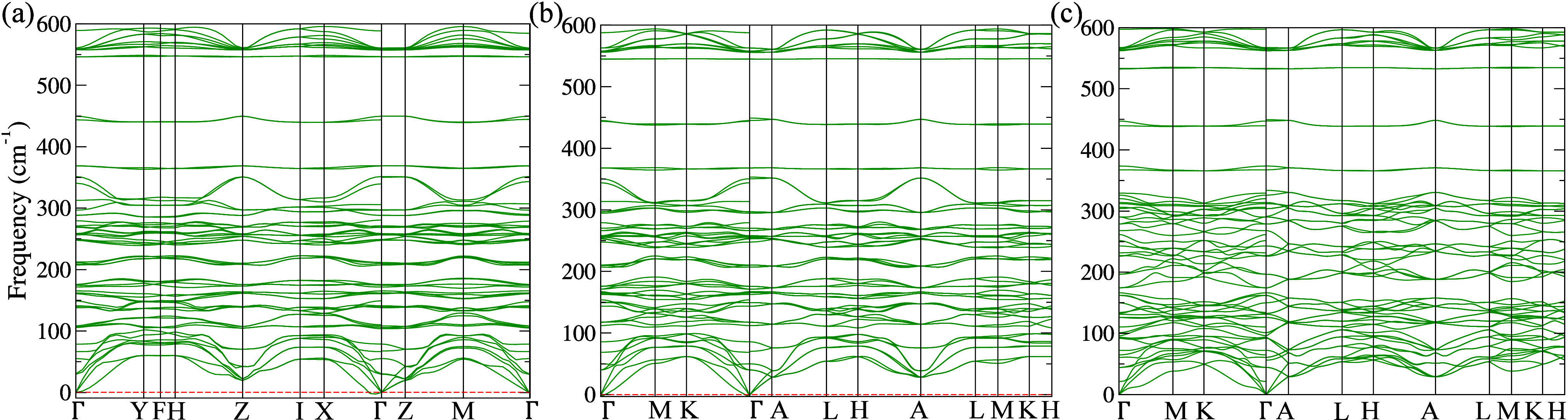
Phonon dispersions calculated with nonanalytical LO-TO corrections for (a) *C*2/*c*, (b) *P*3̅1*c* (in-layer), and (c) *P*3̅1*c* (in-gap) phases.

According to group theory, the irreducible representations of all allowed vibrational modes at the Brillouin zone center (Γ point) for the *C*2/*c* and *P*3̅1*c* phases are defined as follows, respectively:

$$\Gamma_{\mathrm{vib}}^{C2/c}=14A_{g}⊕14A_{u}⊕16B_{g}⊕16B_{u}$$ 6

and

$$\Gamma_{\mathrm{vib}}^{P\mathrm{3̅}1c}=4A_{1g}⊕4A_{1u}⊕6A_{2g}⊕6A_{2u}⊕10E_{u}⊕10E_{g}$$ 7

Out of the total 60 allowed phonon modes (corresponding to 20 atoms per unit cell), three are acoustic modes and the remaining 57 are optical modes. For the monoclinic *C*2/*c* phase, the acoustic modes correspond to *A*_*u*_ ⊕ 2*B*_*u*_, and the optical modes are 14*A*_*g*_ ⊕ 13*A*_*u*_ ⊕ 16*B*_*g*_ ⊕ 14*B*_*u*_. Similarly, for the trigonal *P*3̅1*c* phase, the acoustic modes are given by *A*_2*u*_ ⊕ *E*_*u*_, and the optical modes are 4*A*_1*g*_ ⊕ 4*A*_1*u*_ ⊕ 6*A*_2*g*_ ⊕ 5*A*_2*u*_ ⊕ 9*E*_*u*_ ⊕ 10*E*_*g*_.

For the *C*2/*c* phase, the *A*_*g*_ and *B*_*g*_ modes, and for both *P*3̅1*c* phases, the *A*_1*g*_ and *E*_*g*_ modes are Raman-active modes. In contrast, for *C*2/*c*, the *A*_*u*_ and *B*_*u*_ modes, and for *P*3̅1*c*, the *A*_2*u*_ and *E*_*u*_ modes are infrared-active. All other modes are silent. Figure 4 depicts the calculated frequencies of all the Raman- and infrared-active modes, whereas their numerical values are tabulated in Tables S2–S4. Notably, our calculated frequencies of the Raman-active modes for the *P*3̅1*c* in-layer phase are in good agreement with the experimentally reported data for the same phase by Liang et al.^28^ [see Figure 4(a)].

**Figure 4:**
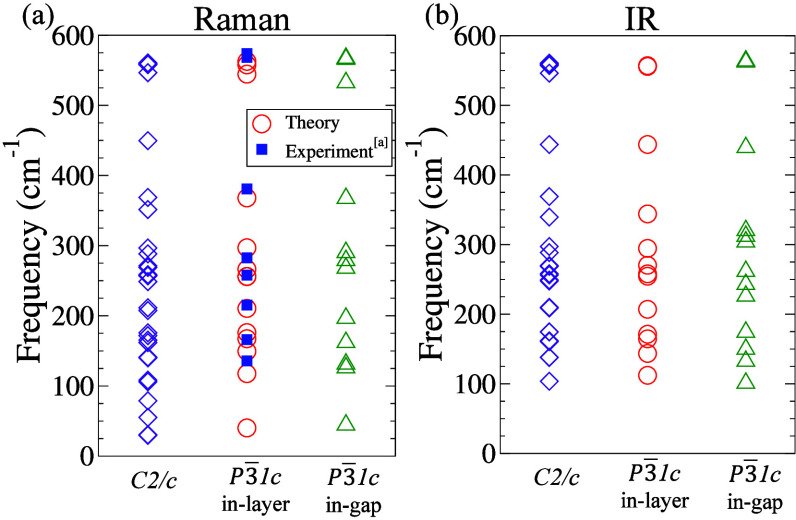
(a) Calculated frequencies of the Raman-active modes, alongside the experimentally reported prominent Raman-active modes of the *P*3̅1*c* (in-layer) phase by Liang et al.^28^ and (b) the infrared (IR) active mode frequencies for the three candidate LiInP_2_S_6_ polymorphs.

#### Charge-Transfer-Function Relationships in Different Phases of LiInP_2_S_6_

To investigate structure–property relationships in LiInP_2_S_6_, we computed the Born effective charge tensors (Z*) for each phase (see Table S5). The results show substantial deviations from the nominal ionic charges (Li^+^, In^3+^, P^4+^, S^2–^). The Z* tensors vary significantly among the studied phases. In the LiInP_2_S_6_ structures, Li exhibits Z* values as high as +1.77*e*, while In reaches up to +4.17*e* in the trigonal phase, with the lowest value found in the monoclinic phase (+2.24*e*). The S atoms attain Z* as low as −2.40*e*, below the nominal −2*e*. Such anomalously large dynamical charges for Li and In (exceeding nominal ionic values) indicate substantial dynamic charge transfer from S to these cations along the In–S and Li–S bonds, reflecting mixed ionic–covalent interactions. By contrast, P displays reduced Z* values (0.73–2.93*e*) relative to its nominal +4*e*, consistent with covalent P–S bonding within the P_2_S_6_ framework. Notably, the largest out-of-plane component (*Z*_zz_^*^) occurs in the *P*3̅1*c* (in-gap) phase; moreover, the out-of-plane Z* of S is significantly higher there than in the *P*3̅1*c* (in-layer) and *C*2/*c* phases, consistent with Li occupying interlayer (van der Waals gap) sites that promote strong out-of-plane Li–S interactions.

### Electronic Properties

In this section, we investigate the electronic structure of the three studied polymorphs of LiInP_2_S_6_. We analyze both the electronic band structure near the Fermi level (*E*_*F*_), calculated along high-symmetry directions in the Brillouin zone, and the orbital-resolved density of states (DOS) (see Figure 5). The band gap is defined as the energy difference between the valence band maximum (VBM) and the conduction band minimum (CBM), where the Fermi level is depicted by a red horizontal dashed line in the band structure plots.

**Figure 5:**
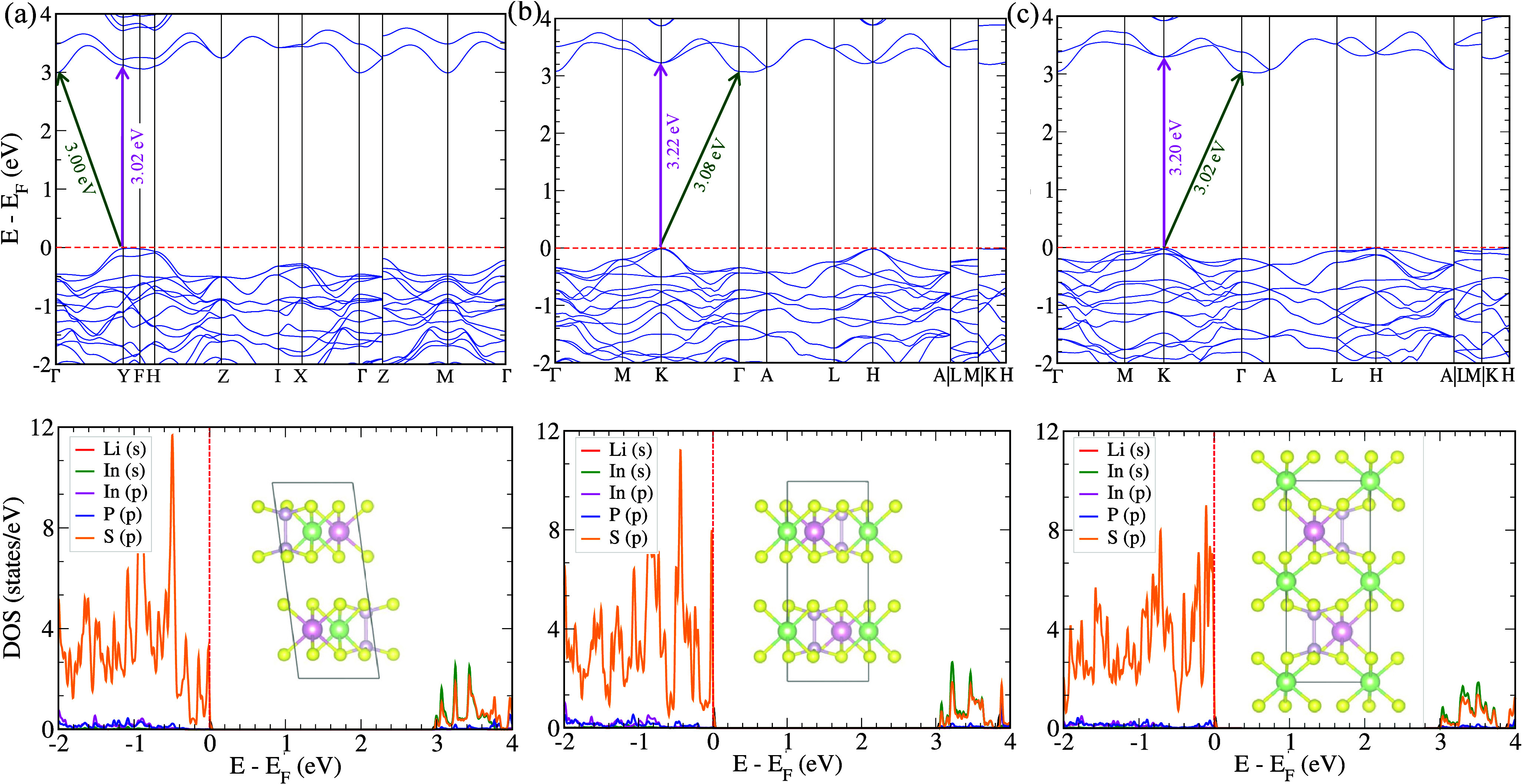
GGA-PBEsol calculated electronic band structures plotted with scissor-operator correction (see main text for details) and the orbital-resolved densities of states (DOSs) obtained using the HSE06 hybrid functional for (a) *C*2/*c*, (b) *P*3̅1*c* (in-layer), and (c) *P*3̅1*c* (in-gap) phases. The green arrows in the band structures indicate the indirect band gaps from the valence-band maximum (VBM) to the conduction-band minimum (CBM), while the pink arrows highlight the direct band gaps in the corresponding phases. The red dashed line marks the Fermi level.

Our results reveal that, within the PBEsol approach, the *C*2/*c* phase exhibits an indirect band gap of 1.88 eV between the Γ and Y symmetry points, while the *P*3̅1*c* (in-layer) and *P*3̅1*c* (in-gap) phases possess indirect band gaps of 1.96 and 1.91 eV, respectively, corresponding to indirect transitions between the K and Γ points (see Figure S2). Notably, for the *P*3̅1*c* (in-layer) phase, Liang et al.^28^ reported a band gap of 1.88 eV using the DFT-PBE method, which is in excellent agreement with our calculations. Since semilocal DFT functionals such as PBEsol typically underestimate band gaps, we employed the HSE06 hybrid functional^47^ to obtain more accurate electronic structures. The HSE06 results yield band gaps of 3.00, 3.08, and 3.02 eV for the *C*2/*c*, *P*3̅1*c* (in-layer), and *P*3̅1*c* (in-gap) phases, respectively. These values are consistent with the 3.00 eV band gap recently reported for monolayer LiInP_2_S_6_ by Bai et al.^30^

To achieve improved agreement with the experimental data and accurately model the optical absorption spectra, we employ a scissor-operator method^49–51^ to correct for the GGA-PBEsol underestimated bandgap. For materials with known accurate band gaps (here obtained from HSE06), the scissor shift Δ_sc_ is defined as

$$\Delta_{\mathrm{sc}}=E_{g}\left(\mathrm{HSE}06\right)-E_{g}\left(\mathrm{PBEsol}\right)$$ 8

Accordingly, the conduction bands of all three phases were rigidly shifted by 1.2 eV, yielding optical properties in closer agreement with experimental observations. Overall as shown in Figure 5, these results indicate that all three stable phases of LiInP_2_S_6_ are indirect-gap semiconductors with band gaps well suited for UV–vis light absorption.

To analyze the orbital contributions to the electronic structure, we examined the density of states (DOS) obtained from both PBEsol calculations (see Figure S2) and those corrected using the HSE06 band gaps by applying the scissor operator (Figure 5). In all studied phases, the significant contribution of the *p* orbitals of S atoms near the Fermi level in the valence band indicates the presence of hole carriers and confirms the *p*-type nature of the LiInP_2_S_6_ materials. Furthermore, a strong hybridization is evident between the S (*p*) orbitals and the In (*s*) orbitals across all studied phases, as demonstrated by their overlap in the conduction band. In comparison with the monoclinic and trigonal phases of CuInP_2_S_6_,^16^ the valence band reveals a strong hybridization between the Cu (*d*) orbitals and the S (*p*) orbitals near the Fermi level, while the conduction band exhibits a similar overlap between the S (*p*) and In (*s*) orbitals. However, in LiInP_2_S_6_, where Li replaced with Cu, the *p* orbital of Li contributes far less near the Fermi level than the *d* orbital of Cu in CuInP_2_S_6_, highlighting the reduced hybridization in LiInP_2_S_6_.

### Optical Properties

The optical properties of a material are influenced by its electronic band structure. Given that LiInP_2_S_6_ exhibits an intriguing band gap range, we were motivated to investigate the optical properties and absorption spectra of the three proposed candidate phases. The optical properties of a solid can be determined through the Kramers–Kronig relations, which connect the real and imaginary parts of the complex dielectric function.^48,61^ The complex dielectric function, *ε*(ω) = *ε*_1_(ω) + *iε*_2_(ω), describes the material’s optical response, where *ε*_1_(ω) represents the real part and *ε*_2_(ω) represents the imaginary part. These components are related through the following expressions:

$$\varepsilon_{1}\left(\omega \right)=1+\frac{2}{\pi }P\int_{0}^{\infty }\frac{\overset{2}{\underset{\alpha \beta }{\sum }}\varepsilon_{2\alpha \beta }\left(\omega^{\prime }\right)\omega^{\prime }}{\omega^{\prime 2}-\omega^{2}+i\eta }\;d\omega^{\prime }$$ 9

$$\varepsilon_{2}\left(\omega \right)=\frac{4\pi^{2}e^{2}}{\Omega }\;\lim_{q\to 0}\frac{1}{q^{2}}\underset{c,v,k}{\sum }2\omega_{k}\delta \left(E_{ck}-E_{vk}-\omega \right)\times \left\langle \mu_{ck+e_{\alpha }q}|\mu_{vk}\right\rangle \left\langle \mu_{vk}|\mu_{ck+e_{\beta }q}\right\rangle$$ 10

Here, *P* denotes the principal value applied to the relation, Ω represents the volume of the crystal, and **q** is the electron momentum operator. The indices *c* and *v* correspond to the conduction and valence bands, respectively, while *w*_**k**_ denotes the *k*-point weights. *E*_*c* **k**_ and *E*_*v* **k**_ are the eigenvalues, and μ_*c* **k**_ and μ_*v* **k**_ are the corresponding wave functions. The **e**_α_ and **e**_β_ are unit vectors along the three Cartesian directions, and ω is the frequency of the electromagnetic radiation. *e* denotes the electron charge, and *ε*_*αβ*_ represents the component of the macroscopic electric field tensor. From the obtained dielectric function, the optical absorption coefficient of the material can be calculated using the following relation:

$$n\left(\omega \right)={\left[\frac{\sqrt{{\varepsilon_{1}}^{2}+{\varepsilon_{2}}^{2}}+\varepsilon_{1}}{2}\right]}^{1/2}$$ 11

In this study, we calculated *ε*_1_(ω) and *ε*_2_(ω) for the three candidates using both PBEsol and HSE06 functionals, with convergence ensured by including a total of 144 bands and employing 8 × 8 × 4 *k*-mesh, as shown in Figure S3. Since the dielectric function depends on the number of conduction bands, we further increased the number of bands from 144 to 200 and varied the *k*-mesh to 16 × 16 × 8. The results confirmed the convergence criteria.

The absorption coefficient quantifies the penetration depth of light into a material. For the *C*2/*c*, *P*3̅1*c* (in-layer), and *P*3̅1*c* (in-gap) phases, as shown in Figure 6, the solid lines represent the HSE06 hybrid functional results, while the dashed lines correspond to the PBEsol calculations. According to the DFT-PBEsol study, the onset of photon absorption occurs within the visible-light range, yet the absorption edges are approximately 0.2 eV higher than the indirect band gaps obtained from the electronic band structure calculations. This discrepancy arises because the optical absorption is governed by the direct band gap, illustrated in Figure 5 with pink arrows, which corresponds to electronic transitions occurring at the same *k*-point. Nevertheless, since all of these materials exhibit an indirect band gap, the photon energy required to excite an electron from the S *p*-orbitals to the In *s*-orbitals is higher than the indirect band gap value.

**Figure 6:**
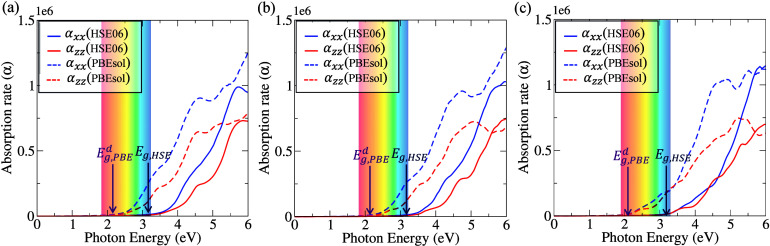
Optical absorption spectra for (a) *C*2/*c*, (b) *P*3̅1*c* (in-layer), and (c) *P*3̅1*c* (in-gap) phases. In all phases, the absorption coefficients satisfy α_*xx*_ = α_*yy*_. Solid lines represent HSE06 results, while dashed lines correspond to PBEsol calculations.

In addition, we examined the optical absorption spectra using the HSE06 hybrid functional for the three candidate phases to obtain a more accurate description of their optical response. The absorption onset for all three structures appears at the UV–vis band edge, consistent with the band gaps calculated from the HSE06 density of states (DOS). These results are in good agreement with the previous study by Bai et al.,^30^ which reported that the optical absorption of monolayer LiInP_2_S_6_ occurs within the UV–vis light range. Furthermore, additional optical properties of the investigated polymorphs, including the refractive index, energy-loss function, and extinction coefficient, which have been extensively examined in related bulk and monolayer structures^62,63^ were analyzed and are presented in Figure S4. Owing to the vdW layered nature of LiInP_2_S_6_, characterized by weak interlayer interactions and strong in-plane covalent bonding, its electronic and optical properties are expected to be sensitive to external strain. Such strain-induced band gap modulation has been widely reported in other two-dimensional materials, including halide 2D perovskites,^64,65^ where biaxial and uniaxial strain have been shown to effectively tune the electronic band gap and enhance optoelectronic performance. These established trends suggest that strain engineering may also provide a promising route for tuning the electronic and optical properties of LiInP_2_S_6_ polymorphs in future studies.

## Conclusions

In this work, we employ comprehensive first-principles calculations to investigate the 2D vdW LiInP_2_S_6_ material. Two previously unreported prototypes, the monoclinic *C*2/*c* and trigonal *P*3̅1*c* (in-gap) phases, are introduced in addition to the experimentally synthesized *P*3̅1*c* (in-layer) phase.^27,28^ The *C*2/*c* phase is identified as the thermodynamic ground state, being 9 meV per unit cell lower in energy than the recently synthesized *P*3̅1*c* (in-layer) phase. AIMD simulations demonstrate that proposed *C*2/*c* and *P*3̅1*c* (in-gap) phases are thermally stable at room temperature. Elastic constant analysis confirms that all three structures are mechanically stable, with the *P*3̅1*c* (in-gap) phase showing the highest stiffness, originating from strong Li–S covalent bonding across the vdW gap. All three candidates exhibit brittle characteristics.

Phonon dispersion calculations further validate their dynamical stability, while Born effective charge analysis reveals a mixed ionic–covalent bonding nature arising from Li–S and In–S interactions. The electronic structures, evaluated using both PBEsol and HSE06 hybrid functionals, show indirect band gaps of 3.00, 3.08, and 3.02 eV for the *C*2/*c*, *P*3̅1*c* (in-layer), and *P*3̅1*c* (in-gap) phases, respectively. As these band gaps lie within the UV–vis range, the calculated optical absorption spectra indicate strong photon absorption in the UV–vis region. These results highlight LiInP_2_S_6_ as a promising material for future optoelectronic applications.

## Supplementary Material



## Notes

Additional details on the Raman/IR active mode frequencies, charge transfer function, DOS from HSE06 functional calculations, and dielectric function calculations on three studied phases (PDF)
