# Supplementary material for: First-Principles Discovery of Novel LiInP2S6 Polymorphs with Promising Optoelectronic Responses
Source: J Phys Chem C Nanomater Interfaces. 2026 Mar 17;130(13):4936–44. doi: 10.1021/acs.jpcc.5c08009 (PMC13051440; doi:10.1021/acs.jpcc.5c08009)
Supplement: Supplementary file 1 [file jp5c08009_si_001.pdf]

Supplementary Information

for

First-Principles Discovery of Novel $LiInP_2S_6$ Polymorphs with Promising Optoelectronic Responses

Pegah Mohammadi[a], **Arjyama Bordoloi**[a], and **Sobhit Singh**[a,b]

[a]Department of Mechanical Engineering, University of Rochester, Rochester, NY 14627, USA

[b]Materials Science Program, University of Rochester, Rochester, NY 14627, USA

Corresponding author: s.singh@rochester.edu

Contents

S1. Optimized Atomic Coordinates S1
S2. Thermal stability S2
S3. Raman- and IR-active mode frequencies for three phases S2
S4. Born effective charges and charge-transfer discussion S4
S5. Electronic properties with PBEsol calculations S5
S6. Dielectric function calculations S5
S7. Optical Properties S6

S1 Optimized Atomic Coordinates

The optimized fractional atomic coordinates for the three investigated prototype structures of $LiInP_2S_6$ (the monoclinic $C2/c$ phase (no. 15), the trigonal $P\bar{3}1c$ in-layer phase (no. 163), and the trigonal $P\bar{3}1c$ in-gap phase (no. 163) are reported in Table S1. All coordinates were obtained from fully relaxed DFT calculations using the PBEsol exchange–correlation functional with D3 van der Waals corrections. The atomic positions are provided as fractional coordinates with respect to the optimized unit cells and are listed according to their corresponding Wyckoff positions.

Table S1: Optimized fractional atomic coordinates of $LiInP_2S_6$ for the studied polymorphs obtained from DFT (PBEsol+D3) calculations.

**Space group**	**Atom**	**Site**	x	y	z
$C2/c$	Li	4e	0.0000	0.1658	0.2499
	In	4e	0.0000	0.4982	0.2499
	P	8f	0.4405	0.6672	0.6631
	S1	8f	0.6540	0.6721	0.1216
	S2	8f	0.7355	0.3286	0.1199
	S3	8f	0.7456	0.5035	0.8798
$P\bar{3}1c$ (in-layer)	Li	2a	0.0000	0.0000	0.2500
	In	2c	0.3333	0.6666	0.2500
	P	4f	0.3333	0.6666	0.6623
	S	12i	0.3240	0.3371	0.8803
$P\bar{3}1c$ (in-gap)	Li	2b	0.0000	0.0000	0.0000
	In	2c	0.3333	0.6666	0.2500
	P	4f	0.3333	0.6666	0.6616
	S	12i	0.3354	0.3447	0.8794

S2 Thermal stability

The temperature range over which a material can endure without undergoing structural distortion is a key property for high-temperature applications. In this context, assessing the thermal stability of the unreported $LiInP_2S_6$ polymorphs, the $C2/c$ and $P\bar{3}1c$ (in-gap) phases, is essential to evaluate their suitability for practical applications. It is worth noting that the $P\bar{3}1c$ (in-layer) phase has been experimentally synthesized, which supports its stability at room temperature.

To this end, *ab initio* molecular dynamics (AIMD) simulations were performed for both polymorphs at 300 K. Figure S1 shows the evolution of the total energy over a 10 ps simulation with a time step of 1 fs, with representative structural snapshots at 0 and 10 ps. The simulations were initiated from the most stable configurations obtained from static DFT calculations, and $2 \times 2 \times 1$ supercells were employed. As shown in Fig. S1, both the $C2/c$ and $P\bar{3}1c$ (in-gap) phases remain structurally intact throughout the simulation, exhibiting no significant energy drift or structural degradation. These results indicate thermal stability at room temperature and supporting their potential applicability in high-temperature iontronic and optoelectronic devices.

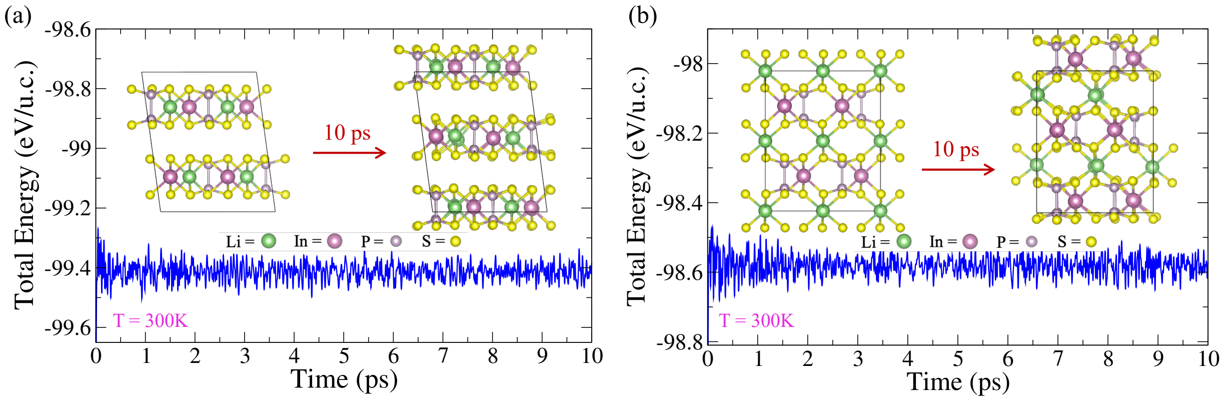

Figure S1: Fluctuations of the total energy during AIMD simulations for the (a) $C2/c$ and (b) $P\bar{3}1c$ (in-gap) phases.

S3 Raman- and IR-active mode frequencies for three phases

As discussed in the main text, the phonon dispersions with non-analytical corrections for all three phases, $C2/c$, $P\bar{3}1c$ (in-layer), and $P\bar{3}1c$ (in-gap), exhibit no imaginary frequencies throughout the Brillouin zone. Therefore, we examine in greater detail the active vibrational modes within their irreducible representations. For the $C2/c$ phase, the A_g and B_g modes are Raman-active, while for the $P\bar{3}1c$ (in-layer and in-gap) phases, the A_{1g} and E_g modes are Raman-active. Correspondingly, the A_u and B_u modes in the $C2/c$ phase, and the A_{2u} and E_u modes in the $P\bar{3}1c$ (in-layer and in-gap) phases, are infrared-active. The mode frequencies for the $C2/c$ phase are listed in Table S2, for the $P\bar{3}1c$ (in-layer) phase in Table S3, and for the $P\bar{3}1c$ (in-gap) phase in Table S4.

Table S2: Raman- and IR-active mode frequencies for the $C2/c$ phase.

Type	Irrep	Frequencies (cm^{-1})
Raman	A$_g$	29.6, 108.2, 140.0, 165.327, 171.6, 211.4, 257.6, 258.7, 268.853, 288.3, 368.7, 546.5, 558.7, 559.5
	B$_g$	30.325, 55.151, 78.8, 105.9, 141.0, 161.8, 175.7, 207.4, 248.6, 256.8, 271.1, 296.7, 351.4, 449.645, 557.9, 560.5
IR	A$_u$	105.4, 137.8, 160.6, 174.4, 208.9, 249.0, 256.3, 269.7, 288.4, 369.0, 546.1, 558.5, 559.5
	B$_u$	66.4, 103.6, 138.1, 161.8, 174.7, 209.9, 247.6, 258.1, 268.7, 297.2, 339.5, 443.7, 557.1, 260.6

Table S3: Raman- and IR-active mode frequencies for the $P\bar{3}1c$ (in-layer) phase.

Type	Irrep	Frequencies (cm^{-1})
Raman	A$_{1g}$	167.3, 255.7, 368.0, 544.5
	E$_g$	40.0, 117.5, 149.4, 175.9, 210.5, 256.1, 266.3, 296.9, 557.7, 562.7
IR	A$_{2u}$	68.2, 164.6, 254.5, 344.0, 443.7
	E$_u$	112.208, 143.622, 171.2, 207.0, 258.0, 270.2, 294.5, 555.8, 556.9

Table S4: Raman- and IR-active mode frequencies for the $P\bar{3}1c$ (in-gap) phase.

Type	Irrep	Frequencies (cm^{-1})
Raman	A$_{1g}$	131.2, 267.9, 367.4, 532.3
	E$_g$	44.39, 91.948, 125.9, 162.0, 196.7, 278.9, 290.3, 565.7, 567.3
IR	A$_{2u}$	21.3, 149.9, 261.6, 320.2, 439.5
	E$_u$	60.8, 101.1, 132.6, 174.3, 226.1, 242.9, 303.9, 312.0, 562.2, 564.0

S4 Charge transfer function in three phases

To investigate the structure–property relationships of $LiInP_2S_6$, we calculated the Born effective charges of atoms in each studied phase, as listed in Table S5. The obtained values deviate from the nominal ionic charges (Li^+, In^{3+}, P^{4+}, and S^{2-}), indicating varying degrees of charge transfer and suggesting a mixed ionic–covalent bonding character. In all three phases, the Li–S and In–S interactions exceed the Madelung limit reflecting the mixed ionic–covalent nature of these interactions, while the P–S bonds exhibit strong covalent character within the P_2S_6 framework. Notably, the largest out-of-plane component (Z^*_{zz}) is observed in the $P\bar{3}1c$ (in-gap) phase, where the Li atoms occupy the interlayer vdW gap and form strong out-of-plane Li–S interactions.

Table S5: Born effective charge tensor components Z^* for $LiInP_2S_6$ in three phases.

		Z^*								
**Phase**	**Ion**	*xx*	*yy*	*zz*	*xy*	*yz*	*zx*	*xz*	*zy*	*yx*
$C2/c$	Li	1.30	1.30	0.63	0.00	0.00	0.01	0.00	0.01	0.00
	In	4.11	4.12	2.24	0.00	-0.01	-0.02	0.00	-0.04	0.00
	P	2.87	2.88	0.90	0.54	-0.02	-0.07	-0.03	0.04	-0.55
	S1	-1.81	-1.90	-0.84	0.62	0.04	0.08	0.18	0.14	0.55
	S2	-1.36	-2.36	-0.77	-0.32	0.13	-0.14	-0.10	0.01	-0.37
	S3	-2.40	-1.31	-0.72	-0.23	-0.13	0.04	-0.06	-0.13	-0.25
$P\bar{3}1c$ (in-layer)	Li	1.28	1.28	0.64	0.00	0.00	0.00	0.00	0.00	0.00
	In	4.17	4.17	2.27	0.00	0.00	0.00	0.00	0.00	0.00
	P	2.91	2.91	0.73	0.52	0.00	0.00	0.00	0.00	-0.52
	S	-1.83	-1.93	-0.73	0.60	0.05	0.07	0.20	0.13	0.56
$P\bar{3}1c$ (in-gap)	Li	1.40	1.40	1.77	0.19	0.00	0.00	0.00	0.00	-0.19
	In	4.16	4.16	2.58	0.00	0.00	0.00	0.00	0.00	0.00
	P	2.93	2.93	0.88	0.55	0.00	0.00	0.00	0.00	-0.55
	S	-1.85	-1.95	-1.02	0.60	0.48	0.16	0.31	0.50	0.59

S5 Electronic properties with PBEsol calculations

The calculated electronic band structures reveal indirect band gaps of 1.88 eV between the Γ and Y symmetry points for the $C2/c$ phase (Fig. S2a), and 1.96 eV and 1.91 eV for the P-31c (in-layer) and P-31c (in-gap) phases, respectively, corresponding to transitions between the K and Γ points in the Brillouin zone (Fig. S2b,c). Furthermore, to analyze the orbital contributions to the electronic structure, we examined the density of states (DOS) obtained from PBEsol calculations for all three phases (Fig. S2).

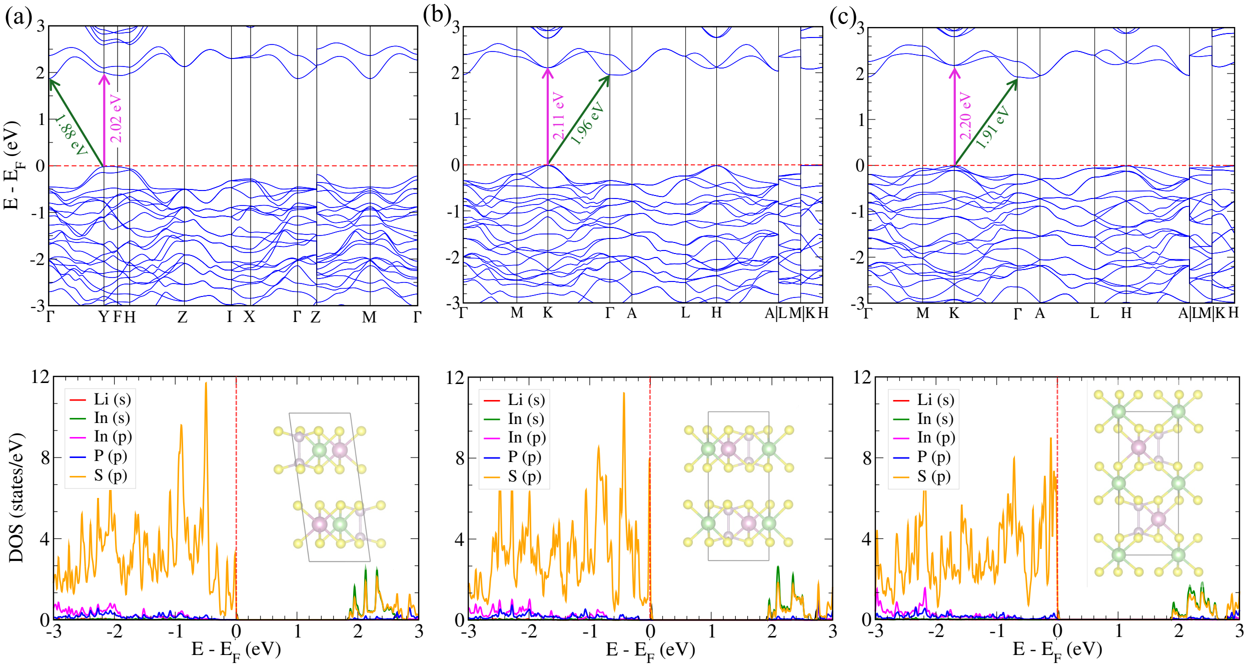

Figure S2: Calculated electronic band structures and orbital-resolved densities of states obtained using PBEsol for (a) $C2/c$, (b) $P\bar{3}1c$ (in-layer), and (c) $P\bar{3}1c$ (in-gap) phases. The green arrows in the band structures indicate the indirect band gaps from the valence-band maximum (VBM) to the conduction-band minimum (CBM), while the pink arrows highlight the direct band gaps in the corresponding phases. The red dashed line marks the Fermi level.

S6 Dielectric function calculations

Since the $LiInP_2S_6$ material exhibits a band gap within the visible spectral range, it is important to investigate its optical properties. As discussed in the main text, the optical response of a solid can be derived from the complex dielectric function, $\varepsilon(\omega) = \varepsilon_1(\omega) + i\varepsilon_2(\omega)$, where $\varepsilon_1(\omega)$ and $\varepsilon_2(\omega)$ represent the real and imaginary parts, respectively. The calculated real and imaginary components are illustrated in Fig. S3.

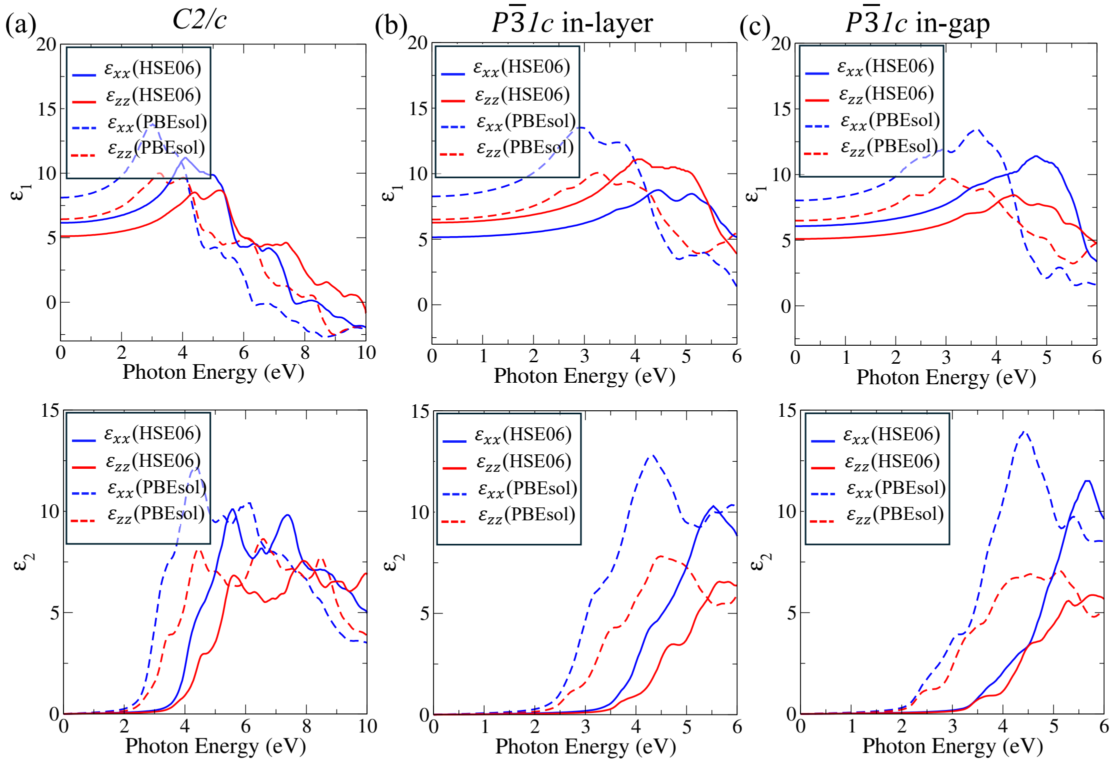

Figure S3: The real ($\varepsilon_1(\omega)$) and imaginary ($\varepsilon_2(\omega)$) parts of the dielectric function for (a) $C2/c$, (b) $P\bar{3}1c$ (in-layer), and (c) $P\bar{3}1c$ (in-gap) phases. In all phases, the dielectric tensor components satisfy $\varepsilon_{xx} = \varepsilon_{yy}$. Solid lines denote HSE06 hybrid-functional results, while dashed lines correspond to PBEsol calculations.

S7 Optical Properties

As discussed in the main text, the refractive index, energy-loss function , and extinction coefficient of $LiInP_2S_6$ were evaluated as functions of photon energy for the three studied prototype structures. These optical quantities were derived from the frequency-dependent complex dielectric function, $\varepsilon(\omega) = \varepsilon_1(\omega) + i\varepsilon_2(\omega)$, calculated using both the PBEsol and HSE06 exchange–correlation functionals.

The refractive index $n(\omega)$ was calculated using

$$n(\omega) = \frac{1}{\sqrt{2}} \left\{ \left[\varepsilon_1^2(\omega) + \varepsilon_2^2(\omega)\right]^{1/2} + \varepsilon_1(\omega) \right\}^{1/2}, \tag{1}$$

while the energy-loss function $L(\omega)$ was evaluated according to

$$L(\omega) = \mathrm{Im}\left[-\frac{1}{\varepsilon(\omega)}\right] = \frac{\varepsilon_2(\omega)}{\varepsilon_1^2(\omega) + \varepsilon_2^2(\omega)}, \tag{2}$$

The extinction coefficient $k(\omega)$ was obtained as

$$k(\omega) = \left\{ \frac{\left[\varepsilon_1^2(\omega) + \varepsilon_2^2(\omega)\right]^{1/2} - \varepsilon_1(\omega)}{2} \right\}^{1/2}. \tag{3}$$

where $\varepsilon_1(\omega)$ and $\varepsilon_2(\omega)$ denote the real and imaginary parts of the dielectric function, respectively.

As shown in Fig. S4(a), the refractive index $n(\omega)$ exhibits pronounced dispersion in the low-energy region for all three phases, followed by a gradual decrease at higher photon energies, with the largest values

occurring in the UV–Vis range. The energy-loss function $L(\omega)$, shown in Fig. S4(b), increases gradually with photon energy and does not display a pronounced plasmon peak, indicating the absence of strong collective plasmon excitations and reflecting the semiconducting nature of $LiInP_2S_6$. Fig. S4(c) presents the extinction coefficient $k(\omega)$, which shows enhanced absorption in the UV–Vis region for all three phases, suggesting potential relevance for optoelectronic and photonic applications operating at high photon energies.

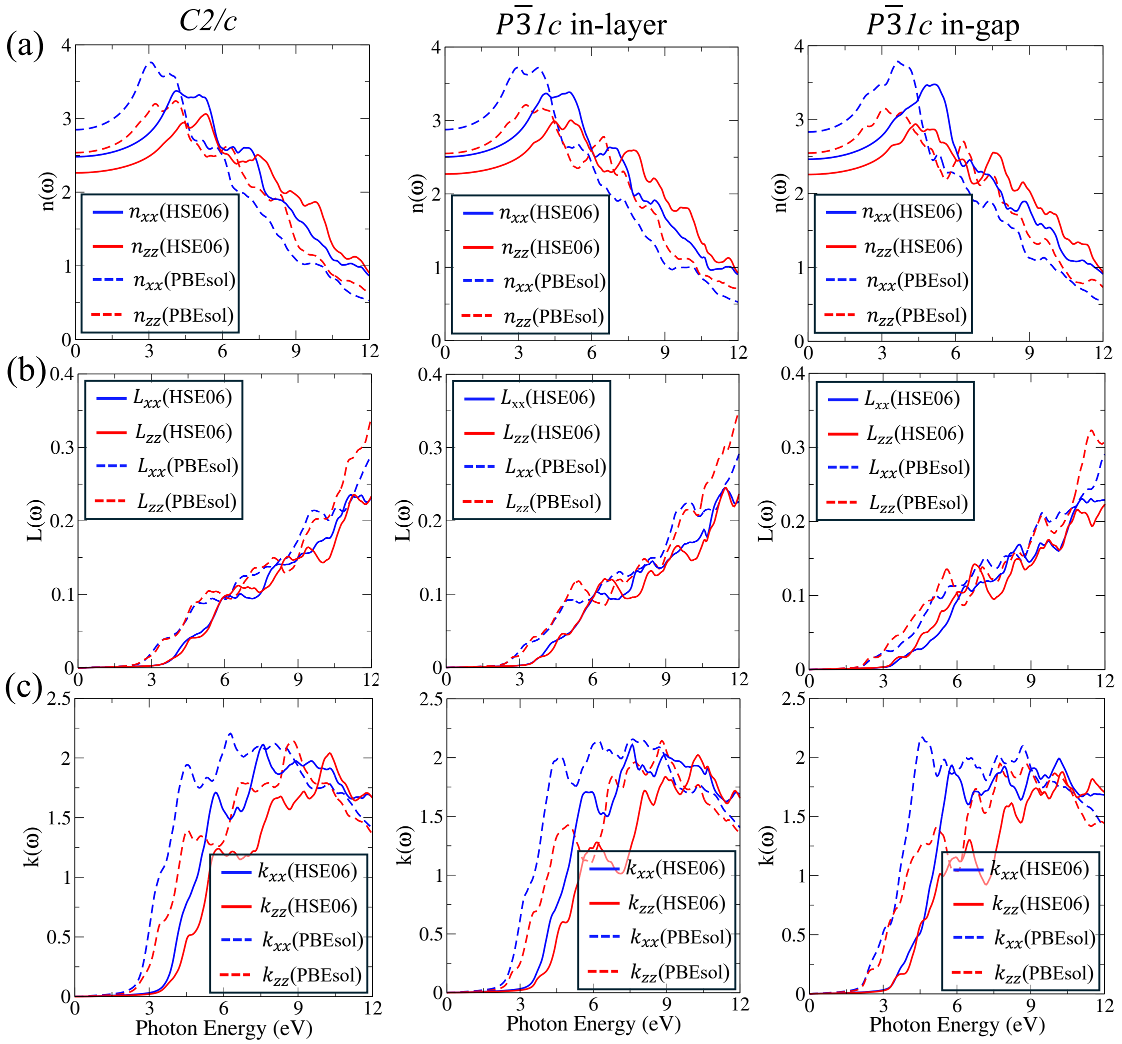

Figure S4: Calculated optical properties of $LiInP_2S_6$: (a) refractive index, (b) energy-loss function, and (c) extinction coefficient for the $C2/c$, $P\bar{3}1c$ (in-layer), and $P\bar{3}1c$ (in-gap) phases. For all phases, the tensor components satisfy $M_{xx} = M_{yy}$. Solid lines represent results obtained using the HSE06 hybrid functional, while dashed lines correspond to PBEsol calculations.
